# WUFlux: an open-source platform for ^13^C metabolic flux analysis of bacterial metabolism

**DOI:** 10.1186/s12859-016-1314-0

**Published:** 2016-11-04

**Authors:** Lian He, Stephen G. Wu, Muhan Zhang, Yixin Chen, Yinjie J. Tang

**Affiliations:** 1 Department of Energy, Environmental and Chemical Engineering, Washington University, St. Louis, MO 63130 USA; 2 Department of Computer Science and Engineering, Washington University, St. Louis, MO 63130 USA

**Keywords:** ^13^C metabolic flux analysis, Energy metabolism, MATLAB, Software

## Abstract

**Background:**

Flux analyses, including flux balance analysis (FBA) and ^13^C-metabolic flux analysis (^13^C-MFA), offer direct insights into cell metabolism, and have been widely used to characterize model and non-model microbial species. Nonetheless, constructing the ^13^C-MFA model and performing flux calculation are demanding for new learners, because they require knowledge of metabolic networks, carbon transitions, and computer programming. To facilitate and standardize the ^13^C-MFA modeling work, we set out to publish a user-friendly and programming-free platform (WUFlux) for flux calculations in MATLAB^®^.

**Results:**

We constructed an open-source platform for steady-state ^13^C-MFA. Using GUIDE (graphical user interface design environment) in MATLAB, we built a user interface that allows users to modify models based on their own experimental conditions. WUFlux is capable of directly correcting mass spectrum data of TBDMS (N-tert-butyldimethylsilyl-N-methyltrifluoroacetamide)-derivatized proteinogenic amino acids by removing background noise. To simplify ^13^C-MFA of different prokaryotic species, the software provides several metabolic network templates, including those for chemoheterotrophic bacteria and mixotrophic cyanobacteria. Users can modify the network and constraints, and then analyze the microbial carbon and energy metabolisms of various carbon substrates (e.g., glucose, pyruvate/lactate, acetate, xylose, and glycerol). WUFlux also offers several ways of visualizing the flux results with respect to the constructed network. To validate our model’s applicability, we have compared and discussed the flux results obtained from WUFlux and other MFA software. We have also illustrated how model constraints of cofactor and ATP balances influence fluxome results.

**Conclusion:**

Open-source software for ^13^C-MFA, WUFlux, with a user-friendly interface and easy-to-modify templates, is now available at http://www.13cmfa.org/or (http://tang.eece.wustl.edu/ToolDevelopment.htm). We will continue documenting curated models of non-model microbial species and improving WUFlux performance.

**Electronic supplementary material:**

The online version of this article (doi:10.1186/s12859-016-1314-0) contains supplementary material, which is available to authorized users.

## Background

Metabolic flux analyses, including flux balance analysis (FBA) and ^13^C metabolic flux analysis (MFA), are widely used to predict or measure in vivo enzyme reaction rates in microbes. FBA can unravel microbial metabolism based on the stoichiometry of the metabolic reactions as well as measurements of the inflow (substrate uptake) and outflow fluxes (biomass and product synthesis). To facilitate the development of genome scale models, much software has been developed [1]. Our research group built a web-based platform named MicrobesFlux (http://www.microbesflux.org/) [2]. This platform can automatically draft a metabolic model from the annotated microbial genome in the KEGG database. Based on users’ feedback, we have re-built our system on a commercial server to improve its functionality, stability, and robustness. The new MicrobesFlux has been updated with both AMPL optimization software and metabolic network information from the latest version of the KEGG database. This platform now includes 3192 species compared to 1304 species in the previous version. Nevertheless, the MicrobesFlux platform still performs only FBA to estimate the flux values. A more rigorous flux analysis requires ^13^C-MFA, which combines FBA with ^13^C isotopic tracing. To complement the current platform, we sought to build an open-source MATLAB-based package (WUFlux) for metabolic flux analysis.

**Fig. 1:**
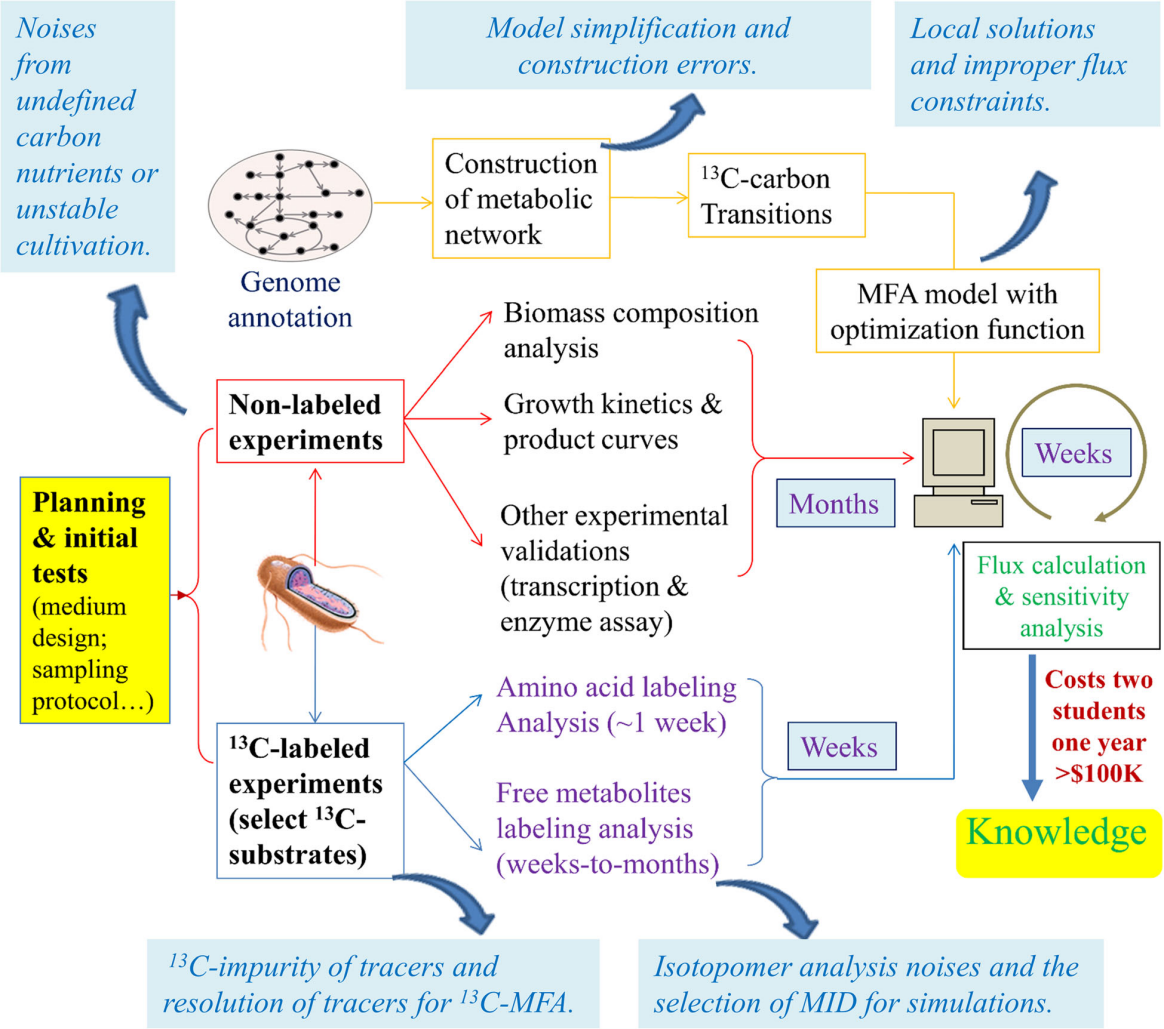
^13^C-MFA protocol and sources of flux analysis variances. In general, ^13^C-MFA of non-model microbial species may require months of work to accomplish [21]. The errors in flux results may come from both experimental measurements and computer modeling. Blue boxes represent the challenges and potential errors generated from ^13^C-MFA procedures

^13^C-MFA requires both experimental and modeling efforts (Fig. 1). ^13^C-labeling experiments consist of feeding the cell culture with defined ^13^C-substrates to fingerprint downstream metabolites with ^13^C-carbons. Once ^13^C has reached a steady state distribution throughout the metabolic network, the labeling patterns of proteinogenic amino acids or free metabolites can be used by a ^13^C-MFA model to decipher the intracellular flux distributions. ^13^C-MFA can help researchers discover novel pathways, resolve reversible and branched fluxes, and quantify circular metabolic routes (e.g., the tricarboxylic acid cycle or TCA cycle). However, ^13^C-MFA is challenging. In terms of experiments, conventional ^13^C-MFA requires that the cell cultures grow in a defined medium and under steady state conditions. The researchers need to select proper ^13^C tracers and obtain high-quality isotopomer data for flux analysis. Meanwhile, construction of the ^13^C-MFA model and flux calculation are demanding for new learners, because they require not only knowledge of metabolic networks and carbon transitions through the pathways, but also computer programming skills (Fig. 1). One ^13^C-MFA project on a non-model microbial species may take two graduate students one year to accomplish. As a matter of fact, fewer than 1000 ^13^C-MFA papers have been published in the past two decades, many of which are reviews or method papers [3]. In addition, most ^13^C-MFA studies focus on several model species (such as *Bacillus subtilis* and *Escherichia coli*). Although there are ~10^9^ microbial species on this planet [4], only a few ^13^C-MFA studies have been carried out on non-model microbial species. If microbiologists had more and better user-friendly and programming-free ^13^C-MFA tools, they could quickly understand diverse microbial metabolisms in a quantitative manner.

To reduce modeling challenges, mass spectrum (MS) data correction tools and ^13^C-MFA software have been developed, including FiatFlux [5], iMS2Flux [6], INCA [7], METRAN [8], OpenFLUX [9], OpenMebius [10], 13CFLUX [11] and 13CFLUX2 [12]. Using these tools and software, researchers can decipher metabolisms of bacterial, plant, and mammalian cells. Our laboratory has also been using ^13^C-MFA extensively to study both model and non-model bacterial species. Based on our experiences, we set out to build an open-source ^13^C-MFA platform (WUFlux) to facilitate analysis of metabolisms in diverse microbes. To reduce the work of constructing flux models, we provide several model templates with predefined metabolic network and carbon atom mappings. As a result, WUFlux can minimize the work done by users and facilitate straightforward flux analysis. Using this platform, we can also standardize and disseminate our MFA work by depositing curated models and flux results into the WUFlux database, which will further benefit the development of fluxomic databases for investigating diverse microbial species [13, 14].

## WUFlux implementation

We chose MATLAB as the programming environment, because it is broadly used by engineers and scientists in both industry and academia. We began with designing a graphical user interface by using GUIDE in MATLAB, and subsequently we created functions directly linked to tables, buttons, pop-up menus, and figures on the user interface.

Constructing a ^13^C MFA model in WUFlux starts with defining the metabolic reactions in the ‘Metabolic Reactions’ section. Instead of asking users to design the metabolic network and carbon transitions from scratch, we have included multiple templates which are suitable for studying chemoheterotrophic (e.g., *E. coli*, *Shewanella oneidensis*, and *Bacillus subtilis*), photomixotrophic cyanobacteria (e.g., *Synechocystis*sp. PCC6803), and vanillin-degrading bacteria (e.g., *Sphingobium* SYK-6) [15–17]. Users can select an appropriate template, and easily make modifications to fine-tune the metabolic network, for example, by knocking out reactions, changing boundary conditions, and adding outflow fluxes.

**Fig. 2:**
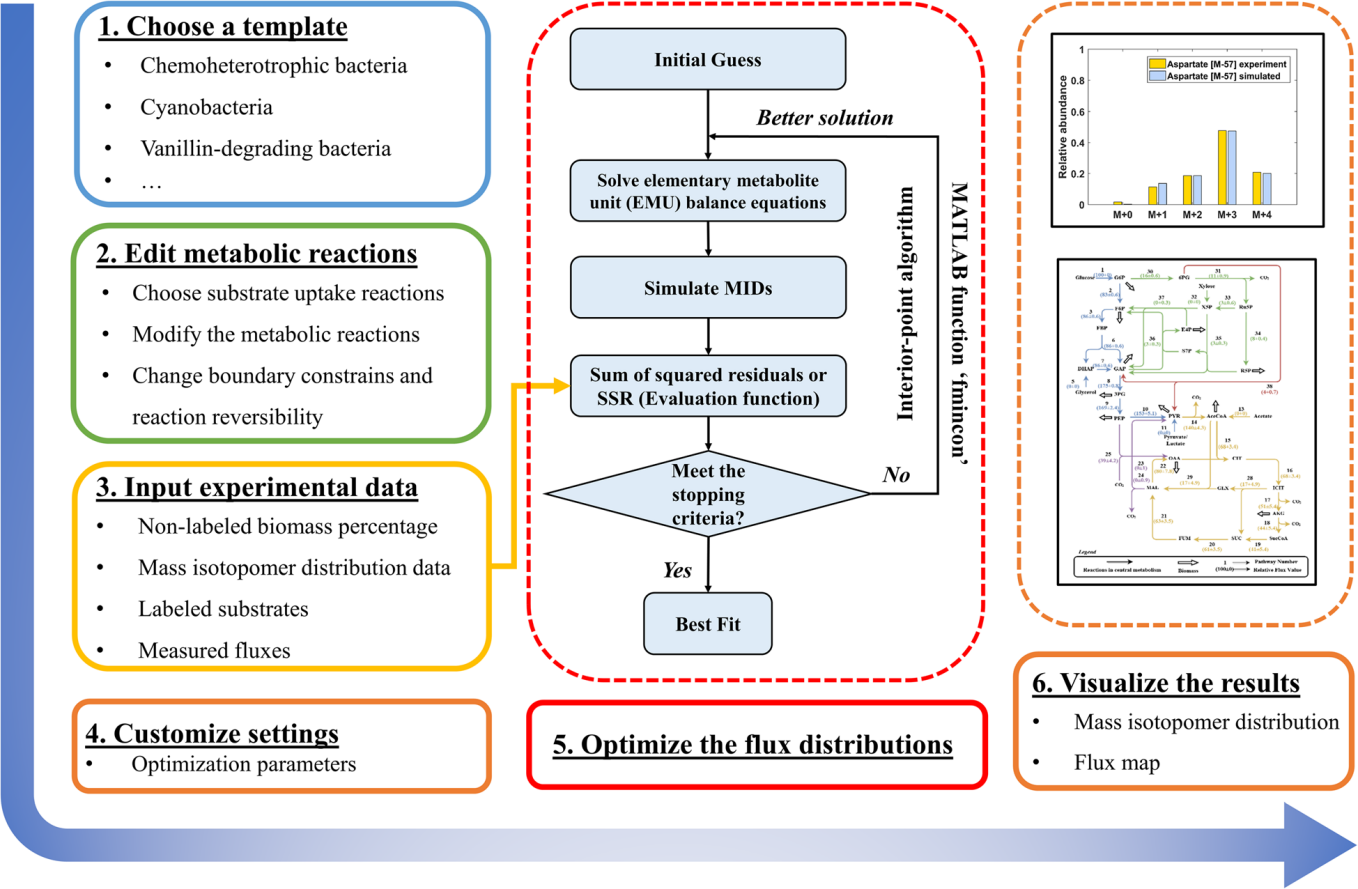
General framework of applying WUFlux for ^13^C-MFA

In the ‘Experiment Data’ section, experimental information must be provided before flux calculations can be made (Fig. 2). The first entry is the ratio of nonlabeled biomass from inoculation to the entire labeled culture. If bacterial inoculation introduces a significant amount of non-labeled biomass in ^13^C-cultures, this ratio (with a default value of 0) will be used to correct the labeling patterns of measured metabolites. Next, the labeling patterns (or the mass isotopomer distribution, MID) of both substrates and proteinogenic amino acids or free metabolites are required. WUFlux can correct raw MSfr(N-tert-butyldimethylsilyl-N-methyltrifluoroacetamide)-derivatized proteinogenic amino acids by employing a previously developed algorithm [18], which promises accurate data correction. In addition, WUFlux can handle the application of multiple tracers (e.g., both glucose and glycerol) or isotopologues (e.g., 50 % [1-^13^C] glucose and 50 % [U-^13^C_6_] glucose) in labeling experiments. The final experimental information is the measured fluxes of any chemicals produced in the cell culture. The measured fluxes will be used in the objective function.

The ‘Settings’ section allows users to customize the optimization parameters (e.g., the number of initial guesses and maximum iteration number). Thereafter, the flux calculation is ready to start. To determine the fluxome, we used the element metabolite unit algorithm [19] to simulate the MIDs of proteinogenic amino acids or free metabolites. This method largely reduces the number of variables compared to the traditional isotopomer mapping matrices approach [11]. The built-in MATLAB function ‘fmincon’ is employed for non-linear optimization, i.e., using ‘interior-point’ as the default algorithm, fmincon minimizes the differences between experimentally and computationally determined data weighted by measured variances. To avoid local solutions, users need to run different initial guesses of fluxes, so that fmincon can find the global optimal solution with the least SSR (sum of squared residuals) (Fig. 2).

The Monte Carlo method is used in the model to determine the confidence intervals of central metabolic fluxes. Briefly speaking, MID data are randomly perturbed with normally distributed noises (within the average range of measurement errors), and the flux profile is then recalculated multiple times, which is customizable in WUFlux. The 95 % confidence intervals, for example, are consequently determined by the upper and lower 2.5 % data via the bootstrap method. Additionally, the *χ* ^2^ test is applied to determine the goodness of fit, which users can use as the reference to determine whether the fitting is statistically acceptable.

Finally, all the flux values and confidence intervals are presented in the ‘Results’ panel, which can be exported to an Excel file. To better present the results, we have included functions that provide direct ways of visualizing the computed fluxes with respect to the constructed metabolic network and visualizing the comparisons between simulated and experimental MID data (see Additional file 1).

## Results and discussion

Figure 2 shows the general procedures for performing ^13^C-MFA with WUFlux: 1) Choose a suitable template, 2) Modify the metabolic network and constraints, 3) Import the experimental data, 4) Customize the optimization parameters, 5) Estimate the flux distribution and determine the confidence intervals, and 6) Visualize the fluxes. More detailed information is provided in Additional file 1.

**Fig. 3:**
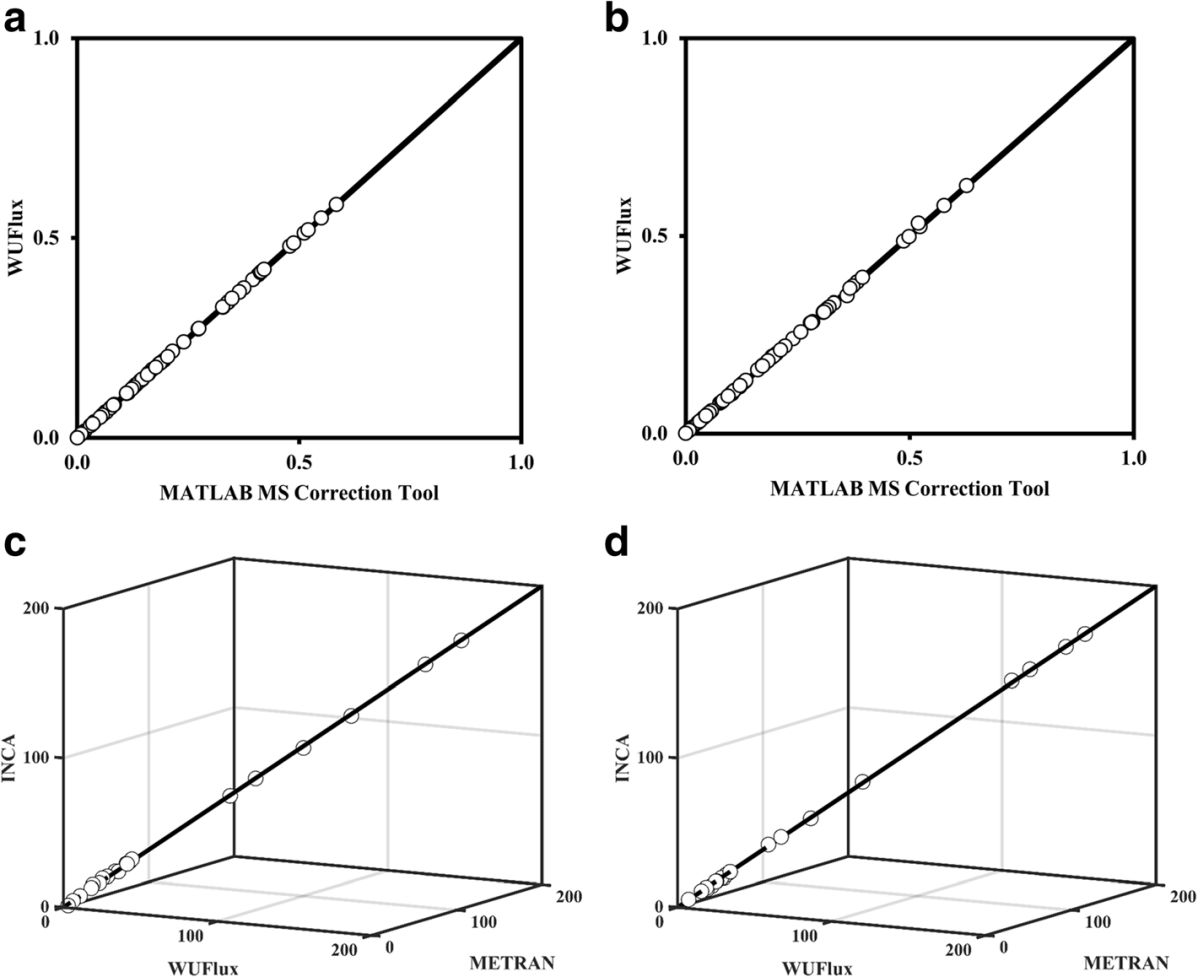
Results validation. The top two figures compare mass isotopomer distribution data determined by WUFlux and mass spectrum correction tool in the control (**a**) and engineered (**b**) *E. coli* strains. The bottom two figures show relative flux distributions determined by WUFlux, METRAN, and INCA in the control (**c**) and engineered (**d**) *E. coli* strains

As a case study, we applied our software to reproduce the MID data and flux profile of both the control and engineered fatty-acid producing *E. coli* strains, which were published in our previous paper [15]. As shown in Fig. 3a-b, WUFlux can convert raw MS data into effective MID data, which is in excellent accordance with MID correction results by a well-developed mass isotope correction software [18]. We further used WUFlux to characterize the fluxomes of *E. coli* strains with corrected MID data. The results were then compared with those generated from METRAN and INCA (Fig. 3c-d and Additional file 2: Table S1). In general, the estimated flux values as well as the major changes between the control and engineered strains agree well with published data and optimization results from other software. All the differences are within 2 % of the glucose uptake rate. The flux results may differ for several reasons (Fig. 1). First, different software may employ different optimization algorithms and solvers for flux calculations. For example, WUFlux relies on the MATLAB built-in function ‘fmincon’, while INCA employs MATLAB’s ‘lsqlin’ function. Second, MID data used for flux calculation are not identical (e.g., WUFlux did not select the MID data of proline because this amino acid often shows high noise-to-signal ratios). Third, the detailed model settings (e.g., the objective functions, biomass equations, statistical analysis, and flux constraints) may not be exactly the same among those software. Additionally, we want to point out the flux calculations can differ between cases with and without consideration of isotopic impurity of labeled substrates [20] and natural abundance of nonlabeled carbons (Additional file 2: Table S1). To gain a more accurate flux analysis, we recommend users to consider both effects.

**Fig. 4:**
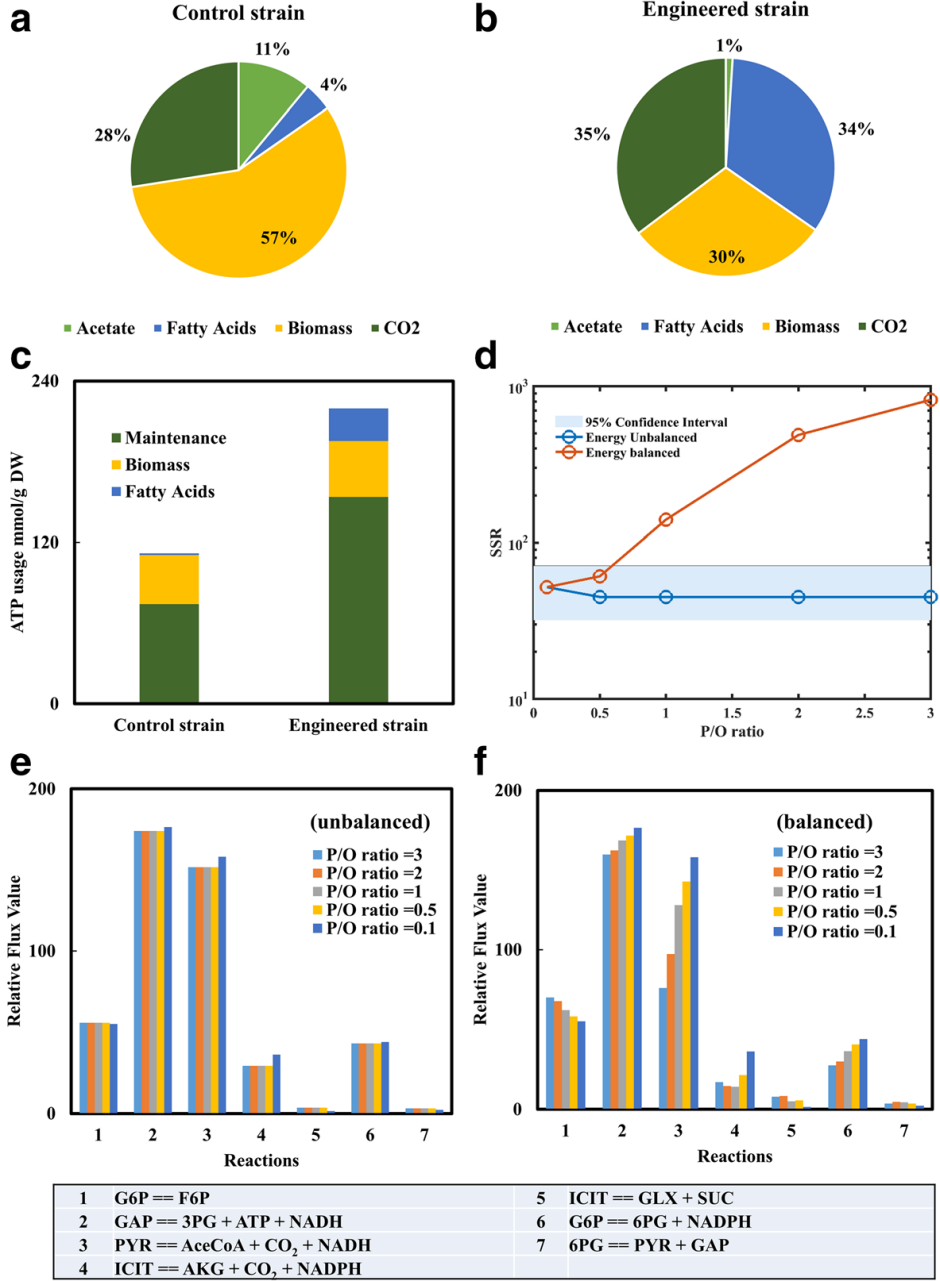
Carbon and energy distributions in both control and engineered *E. coli* strains. **a** carbon fates in the control strain; **b** carbon fates in the engineered strain; **c** ATP usage for biomass, fatty acids, and maintenance loss; **d** the influence of P/O ratios on SSR; **e** the influence of P/O ratios on flux calculation under ‘energy unbalanced’ conditions; and **f** the influence of P/O ratios on flux calculation under ‘energy balanced’ conditions. ‘Energy balanced’ represents the condition when the ATP maintenance loss is assumed as zero, and ‘energy unbalanced’ represents the condition when the ATP maintenance loss is unconstrained. The relative flux values in figures **e** and **f** are normalized to a glucose uptake rate of 100. Abbreviations for metabolites: 3PG, 3-phosphoglycerate; 6PG, 6-phosphogluconate; AceCoA, acetyl-CoA; AKG, α-ketoglutarate; F6P, fructose 6-phosphate; G6P, glucose 6-phosphate; GAP, glyceraldehyde 3-phosphate; GLX, glyoxylate; ICIT, isocitrate; PYR, pyruvate; and SUC, succinate

^13^C-MFA is an important tool to reveal a cell’s energy state for cell biosynthesis and well-being. In cellular processes, the energy molecule ATP is not only used for biosynthesis, but also consumed for diverse non-growth associated activities, such as cell repair and stress responses. ^13^C-MFA can calculate the total ATP generation from catabolism and ATP consumption for biosynthesis. The excess ATP can be assumed to be the maintenance cost, which is defined as the overall ATP required for maintaining each gram of biomass (mmol/g DW) in this study. Here, we demonstrate how to apply WUFlux to study energy metabolism by using the isotopomer data from reference [15]. In Fig. 4a, we divided the carbon distributions into biomass synthesis, fatty acid production, CO_2_ loss, and acetate production. The results prove that the engineered strain can successfully direct more carbon flow towards fatty acid production, while the control strain uses the majority of the carbons for biomass synthesis. Additionally, we can use flux data to analyze cellular energy expenditure. For example, ATP loss for maintenance energy in the engineered strain was estimated to be two-fold larger than that in the control strain (Fig. 4b-c), suggesting that overproduction of fatty acid led to a higher energy burden on the host strain. ^13^C-MFA can quantify cell energy fluxes and is particularly useful for understanding the ATP and cofactor balances in engineered microbial hosts. Lastly, users can add an ‘energy balance’ equation in WUFlux (e.g., the ATP net production is equal to consumption for biosynthesis). Under such an assumption, the P/O ratios may impact flux calculation results. Figure 4d-f illustrates the influence of P/O ratios on flux estimation of the engineered *E. coli* strain. The results show that flux estimation is insensitive to P/O ratios under ‘energy unbalanced’ conditions (when the flux towards ATP maintenance loss is unconstrained, Fig. 4d and e). However, the flux values of many pathways and the values of SSR can be significantly affected by the P/O ratio under ‘energy balanced’ conditions (when the ATP maintenance loss is assumed to be zero, Fig. 4d and f).

## Conclusion

^13^C-MFA is a powerful tool for metabolism analysis, but the overall process of performing ^13^C-MFA is usually not fast enough for biologists to characterize novel microbial species or to provide timely insights into engineered strains in the design-build-test-learn cycle. To overcome this problem, we have designed an open-source MATLAB-based platform, WUFlux, which provides programming-free and straightforward ways of performing ^13^C-MFA. By testing WUFlux against the other software, we showed that WUFlux can correct raw MS data and reproduce the flux estimation of previously published flux analysis studies. Because the MATLAB codes of all function files in WUFlux are open to researchers, users can extend or enhance its capabilities. By using this platform, we can standardize and document the details of ^13^C-MFA studies. We will continue to update the software package by including more flux models of non-model microbial species.

## Availability and requirements

- Project name: WUFlux
- Project homepage: www.13cmfa.org
- Operating systems: Preferably Windows OS 7 or higher
- Programming language: MATLAB
- Other requirements: MATLAB 2012b or higher with optimization toolbox, symbolic math toolbox, and statistic toolbox.
- License: WUFlux is freely available.
- Any restrictions to use by non-academics: none

## Additional files

Additional file 1: User manual for WUFlux (available at www.13cmfa.org). (PDF 1218 kb)
Additional file 2: Comparison of flux estimations from WUFlux, METRAN, and INCA. (DOCX 18 kb)
