# Supplementary material for: WUFlux: an open-source platform for 13C metabolic flux analysis of bacterial metabolism
Source: BMC Bioinformatics. 2016 Nov 4;17:444. doi: 10.1186/s12859-016-1314-0 (PMC5096001; doi:10.1186/s12859-016-1314-0)
Supplement: Additional file 1: — User manual for WUFlux (available at www.13cmfa.org). (PDF 1218 kb) [file 12859_2016_1314_MOESM1_ESM.pdf]

WUFlux User Manual
Version 1.0

You are welcome to contact us for advice or questions about this WUFlux user manual. Your feedbacks will help us improve our platform and update our model systems.

Lian He, E-mail: lianheenvi@gmail.com

Yinjie J. Tang, E-mail: yinjie.tang@wustl.edu.

Table of Contents

MATLAB Requirements 1

1 Installation 1

2 General procedures for using WUFlux 1

2.1 Open WUFlux 1

2.2 Start a new ^{13}C MFA model 2

2.2.1 Substrate uptake reactions 2

2.2.2 Reactions in the central metabolism 3

2.2.3 Linear equality and inequality constraints 3

2.3 Input experimental data 3

2.3.1 Nonlabeled biomass percentage 4

2.3.2 Mass isotopomer distribution 4

2.3.3 Labeled substrates 5

2.3.4 Measured fluxes 6

2.4 Customize optimization settings 6

2.5 Calculate flux distributions 6

2.6 Visualize the results 7

Appendix 1. Description of files in WUFlux 11

Appendix 2. Abbreviations for metabolites 12

MATLAB Requirements

To use this software, you need MATLAB 2012b or a higher version, together with optimization toolbox, symbolic math toolbox, and statistic toolbox.

1 Installation

WUFlux is available at http://13cmfa.org/ or http://tang.eece.wustl.edu/ToolDevelopment.htm. Click the icon ‘WUFlux’ to download the software.

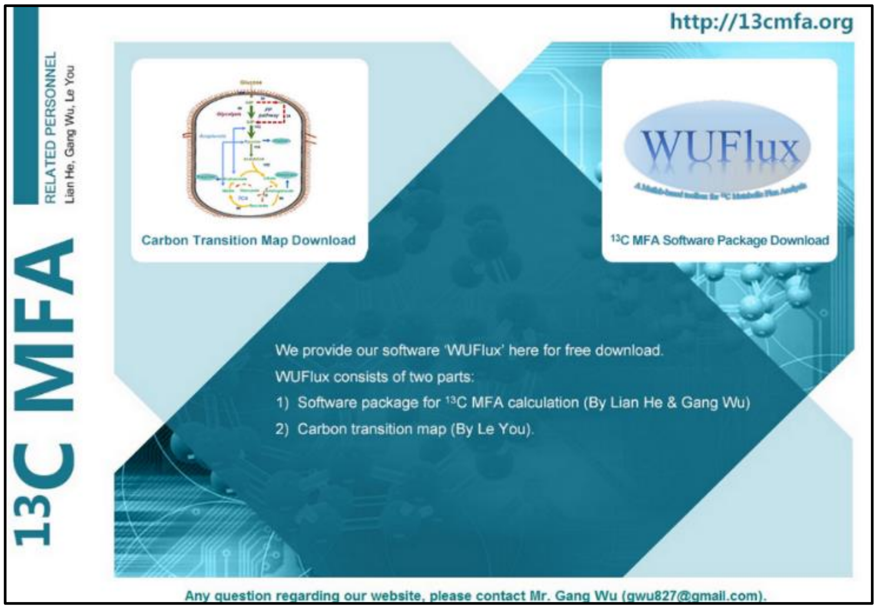

After the download is finished, unzip the package, and then add the folder into the MATLAB working directory. Open ‘Set Path’ dialog box by typing ‘pathtool’ in the command window, add WUFlux folder in MATLAB search path, and then save the change.

2 General procedures for using WUFlux

2.1 Open WUFlux

Simply type ‘WUFlux’ in the command window, or open WUFlux folder, right click WUFlux.m and then choose ‘Run’. The WUFlux interface will appear. The user interface differ slightly according to the screen resolution and operating system of your computer. All the following screenshots were taken on a PC equipped with the Windows 10 operating system and a screen resolution of 1980×1020 pixels.

On the user interface, there are five buttons at the top: Metabolic Reaction, Experimental Data, Run, Results, and Settings. Their functions will be described in the following sections. The buttons at the bottom allow you to start a new project, open a previous project, rename a model, and export current results into an Excel file.

2.2 Start a new ^{13}C MFA model

To build an MFA model, click 'Metabolic Reactions'. Then, in the '*Edit*' panel, select a template from the three choices: chemoheterotrophic bacteria, cyanobacteria, and vanillin-degrading bacteria templates. Key reactions in the central carbon metabolism will appear. You can modify the template by knocking out certain reactions, changing the lower and upper bounds, and defining the reaction reversibility. All the changes made in WUFlux will be automatically saved in the model, so you don't need to save the project from time to time. The carbon transition rules are presented in the last column of the table, which are already defined in the model and are not subject to change.

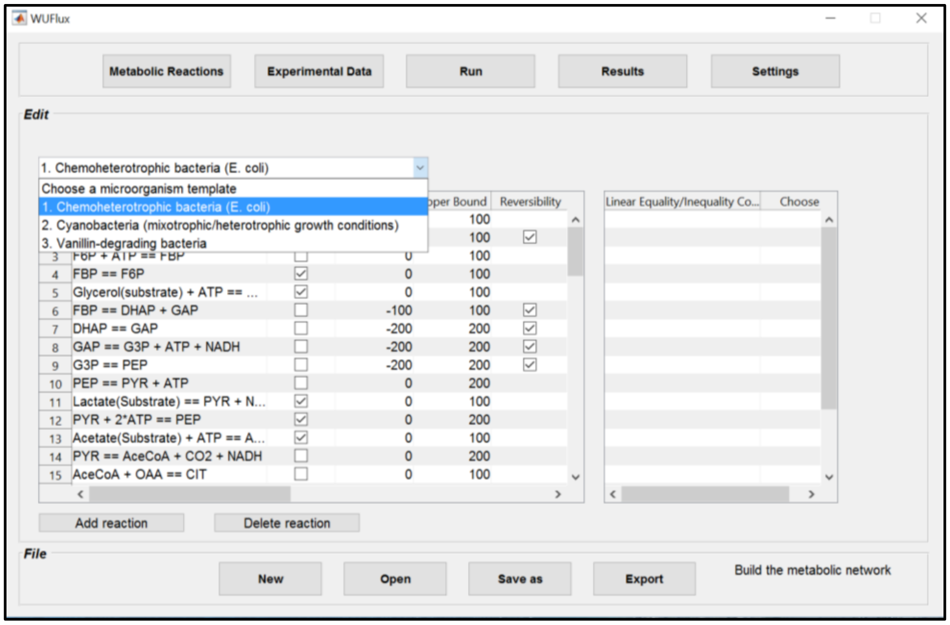

2.2.1 *Substrate uptake reactions*

In the metabolic reactions shown on the interface, choose one or multiple substrates according to your experimental design by unchecking the 'knock out' boxes and giving reasonable ranges. In each template, there are several substrate uptake reactions for glucose, xylose, acetate, pyruvate, glycerol, $NaHCO_3/CO_2$, C1 unit, and OAA. The last two substrates are used for only the vanillin-degrading bacteria that can convert vanillin into pyruvate, oxaloacetate, and methyl-THF. If applying the template for vanillin-degrading strains, you need to define the labeling patterns of those three metabolites instead of vanillin itself. In each template, the labeling pattern of $NaHCO_3/CO_2$ is used to define the labeling pattern of the ambient CO_2. The following table shows which substrates can be used in each template.

Templates	Substrates
**chemoheterotrophic bacteria**	glucose, xylose, acetate, lactate/pyruvate, glycerol
**mixotrophic/heterotrophic cyanobacteria**	glucose, acetate, lactate/pyruvate, glycerol
**vanillin-degrading bacteria**	vanillin (OAA+PYR+C1)

2.2.2 *Reactions in the central metabolism*

WUflux provides all the reactions in the central carbon metabolism, including the Embden–Meyerhof–Parnas pathway, the oxidative pentose phosphate pathway, the Entner–Doudoroff pathway, the TCA cycle, the Calvin cycle (only in the cyanobacterial template), the C1 (including 5-methyl-tetrahydrofolate and 5,10-methylene-tetrahydrofolate) metabolism pathway, and the anaplerotic pathway. Amino acids synthesis pathways are also included in the model. The abbreviations for intracellular metabolites are listed in Appendix 2. The chemoheterotrophic-bacteria template also includes cofactor balance which can be used for estimating NAD(P)H and ATP consumption and production in the central carbon metabolism.

To add to the metabolic network, add reactions related to product excretion by clicking the button ‘Add reaction’. Type in the reaction on the blank line at the bottom. To delete the reaction you just added, click ‘Delete reaction’. Please **note** that a new reaction in the central metabolism cannot be added in the template, and all the carbon transition fates have been pre-defined.

WUFlux uses a MATLAB built-in function, ‘equationsToMatrix’, to convert linear equations to the corresponding matrix form. You can refer to the following website, http://www.mathworks.com/help/symbolic/equationstomatrix.html?refresh=true, for more information regarding how to modify the reactions. The intracellular metabolites are treated as variables in the model.

Here are some examples showing how to correctly write reactions:

Correct Expressions	Incorrect Expressions	Problems
PEP + 2*ATP == PYR	PEP + 2ATP == PYR	‘*’ is missing in the equation.
	PEP + 2*ATP => PYR	‘==’ should be used in the equation, while the reversibility is defined elsewhere in the table.
	PEP + 2ATP == pyruvate	‘PYR’ should be used in the model. (See Appendix 2 for abbreviations for all the metabolites used in WUFlux).

2.2.3 *Linear equality and inequality constraints*

Additionally, you can add linear equality/inequality constraints. The following table shows several examples.

Constraints	The Constraint Expressions
$2<v_2<10$	$v_2-10<0$<br>$2-v_2<0$
$v_2-v_1+3*v_3=9$	$v_2-v_1+3*v_3-9=0$

WUFlux automatically treats v_1, v_2 as flux variables, and the numbers assigned match those in the table of Metabolic Reactions.

2.3 Input experimental data

To import experimental data, click the button ‘Experimental Data’. Two types of experiment data are required: the mass isotopomer distributions of metabolites and labeled substrates. Two data types are optional: the nonlabeled biomass percentage and the measured fluxes.

2.3.1 *Nonlabeled biomass percentage*

In tracer experiments, the inoculation ratio of the non-labeled seed culture to the labeled medium needs to be maintained below 0.5% (v/v) so as to minimize the non-labeled carbons in the final biomass samples. However, in certain circumstances, this inoculation ratio has to be high, for example, to reduce the culture lag phase. In such cases, correct isotopomer data to remove ^{12}C noise caused by inoculation, and recalculate the ratio of non-labeled biomass from the inoculation to the final labeled culture (at the time of harvest):

$$ratio = \frac{OD_{seed\_culture} \cdot V_{inoculation}}{OD_{labeled\_culture} \cdot V_{labeled\_culture}} ,$$

where *OD* represents the optical density, and *V* represents the volume. The default value is 0. WUFlux will now use the nonlabeled biomass percentage to correct the isotopomer data based on the equation below by:

$$MID_{\text{corrected}} = \frac{MID_{\text{exprimental}} - ratio \cdot MID_{\text{natural}}}{1 - ratio} ,$$

where '*natural*' represents the labeling patterns of natural metabolites.

2.3.2 *Mass isotopomer distribution*

Mass isotopomer distribution (MID) data are required for calculating fluxes. In the current version, you can use the MIDs of 14 proteinogenic amino acids and 10 free metabolites in the central metabolism to optimize flux distributions.

You have **three options** to load mass isotopomer distribution (MID) data into WUFlux:

1. Correct your MID data using the software at http://tang.eece.wustl.edu/ToolDevelopment.htm or other correction tools. Manually type in all the corrected MID data, and check the boxes of MIDs that are chosen for simulations.
   Options 2 and 3 use the Excel templates provided in the package. Simply copy your data into the appropriate template file.
2. Click the button 'Load MID data', and then select the Excel file 'MIDtemplate_corrected' containing the corrected MID data.
3. Click 'Convert Raw MS Data', and select the Excel file 'MIDtemplate_uncorrected' containing the raw MS data. If more than two sets of data from the same experiment are provided, WUFlux automatically calculates the average values and standard deviations.

The Excel templates are provided in the package, and you can simply copy your data and then paste them into those files.

In the following figure, WUFlux has checked the MID data and says that the data for Alanine $[M\text{-}57]^+$ is not right, because the summation of M+0, M+1, M+2, and M+3 is not equal to one. Under this circumstance, you should verify your data. Also, you may encounter negative MID

data. Usually, the number is close to zero (e.g., -2×10^{-5}), and you can change that number to 0. If, however, the number is not close enough to zero, you should examine your MID data again.

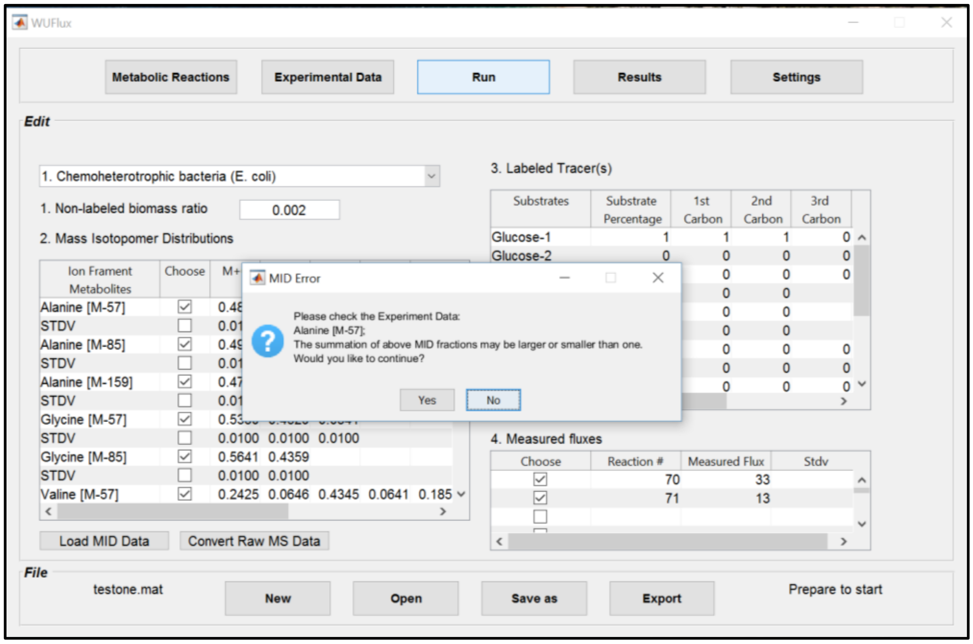

2.3.3 Labeled substrates

On the right side of the '*Edit*' panel, you can define the substrates used in the ^{13}C labeling experiments. Here, only the ^{13}C ratio of each carbon atom in the substrate needs to be defined. For example, [1, 2-^{13}C] glucose can be expressed as [1, 1, 0, 0, 0, 0], which denotes that the first two carbons are labeled as ^{13}C-carbons and the rest as ^{12}C-carbons. You do not need to consider the labeling information of other substrates if they are not applied in your model, since their uptake reactions have been 'knocked out'.

In the current version, up to three isotopologues of one substrate can be included in the model. For instance, if you use 30% natural glucose and 70% [1, 2-^{13}C] glucose in your experiment, define both the percentages and the MID of each isotopologue as follows:

**Substrates**	**Substrate Fractions**	**1st Carbon**	**2nd Carbon**	**3rd Carbon**	**4th Carbon**	**5th Carbon**	**6th Carbon**
**Glucose_1**	0.3	0	0	0	0	0	0
**Glucose_2**	0.7	1	1	0	0	0	0
**Glucose_3**	0	0	0	0	0	0	0

Note that the sum of substrate fractions must equal to one.

It is recommended to take into account the natural abundance of ^{13}C, i.e., put '0.0107' instead of '0' for nonlabeled carbon atoms. Also, consider the isotopic purity of ^{13}C-labeled carbon if such information is available.

2.3.4 Measured fluxes

If any chemicals are produced in large quantities, you should measure their concentrations over time and calculate the effluxes in mmol metabolites produced per gram of dried biomass per hour (i.e., mmol/g DW/h). The following is an example of defining the measured fluxes.

Choose	Reaction #	Measured Flux	STDV
☑	70	13	3
☑	71	36	5
☐	0	0	0

The reaction numbers match those in ‘Metabolic Reactions’. Please note that if relative flux values (which are normalized to, for example, the glucose uptake rate) are used in ‘Metabolic Reactions’, then both the measured flux and standard deviations should also be converted into the relative values as well.

2.4 Customize optimization settings

Before calculating the fluxes, you can modify the parameters in the ‘Settings’ panel.

The MATLAB built-in function ‘fmincon’ solves the nonlinear optimization problem. The parameters, TolFun, TolCon, TolX, MaxIter, and MaxFun, have the same meanings as described on the website: http://www.mathworks.com/help/optim/ug/fmincon.html. The default algorithm of the ‘fmincon’ solver is ‘interior-point’. To get the global optimum, 10 or multiple initial guesses are recommended.

In addition, the Monte Carlo method is employed to calculate the confidence interval of fluxes. In this method, WUFlux perturbs the measured data with normally distributed noises, whose averages (μ) are zero, and standard deviations (δ) are based on the experimental results. However, δ values are assumed to be no less than the ‘minimum standard deviation of the measured MIDs’, whose default value is 0.01.

2.5 Calculate flux distributions

Basically, fluxes are determined by minimizing the following objective equations in WUFlux:

$$\sum_{i=1}^{n}\left(\frac{MID_{\mathrm{exp,i}}-MID_{\mathrm{sim,i}}}{\delta_{\mathrm{exp,i}}}\right)^2+\sum_{j=1}^{m}\left(\frac{v_{\mathrm{exp,j}}-v_{\mathrm{sim,j}}}{\delta_{\mathrm{exp,j}}}\right)^2,$$

where ‘*exp*’ represents the experimental data and ‘*sim*’ the simulated values.

In addition, the following equations are applied in WUFlux to determine the forward and backward fluxes of a reversible reaction:

$$v_{foward} = v^* - \min(-v_{net}, 0),$$

$$v_{backward} = v^* - \min(v_{net}, 0),$$

$$v^* = \beta \frac{v_{reversibility}}{1 - v_{reversibility}},$$

where β is 100, and $v_{reversibility}$ is within [0, 0.9999]. The maximum value of v^* is roughly 10^6. WUFlux first calculates v_{net} (the net flux of a reaction) and $v_{reversibility}$ (the reversibility coefficient), and then transforms the values of v_{net} and $v_{reversibility}$ into the values of $v_{forward}$ and $v_{backward}$.

To calculate the intracellular flux distributions, click ‘Run’, and the following window will appear to show the progress of the on-going calculation. All the flux calculations starting from each initial guess will be stored in the file.

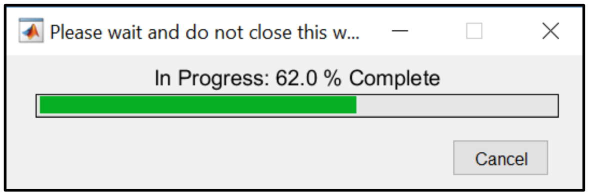

Once finished, another window will appear:

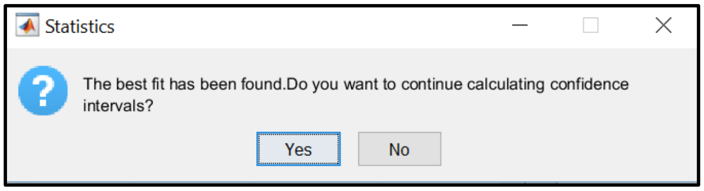

Either click ‘Yes’ to continue calculating confidence intervals, or click ‘No’ to stop the calculation for the time being. The ‘Start’ button changes to the ‘Continue’ button, and you can resume calculating confidence intervals later.

2.6 Visualize the results

Click ‘Results’ to see the best fit of flux distributions, confidence intervals, and standard deviations. A figure showing the correlation of measured and simulated MID data will be automatically generated on the right side. Additionally, the χ^2 test can be applied to determine the goodness of fit (shown at the bottom line), which you can use as a reference to determine whether the final fitting is statistically acceptable. The interface also has a ‘Remove results’ button, which allows you to remove the current flux results. However, once deleted, the results cannot be recovered.

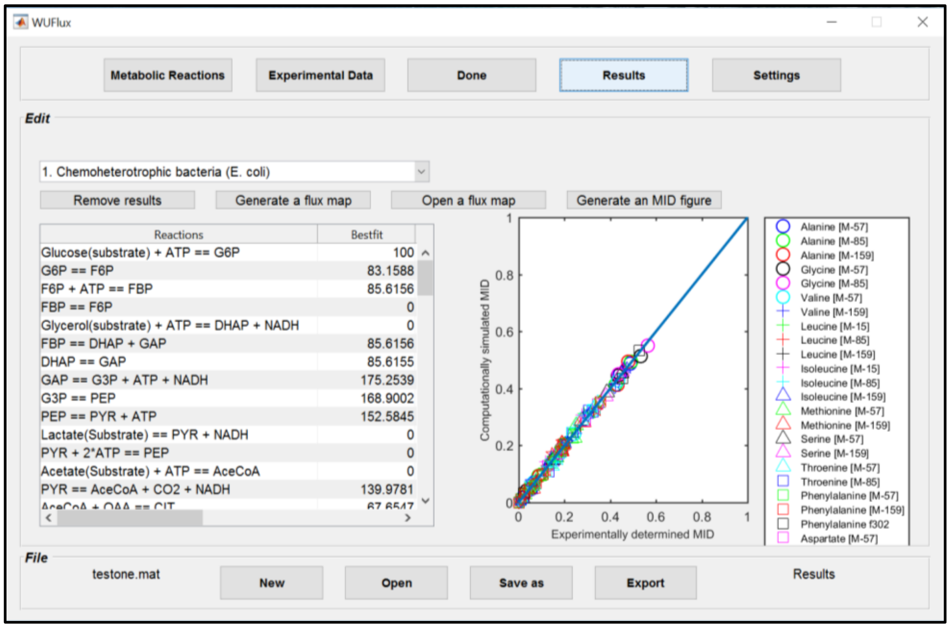

To visualize the flux results with respect to constructed metabolic network, click the button ‘Generate a flux map’. The file is saved in the svg format, which is displayed in the MATLAB built-in web browser and can also be read by other browsers (e.g. Chrome, Firefox, and Edge). The svg files can be loaded into some other online software (e.g., draw.io), which enables conversion from the svg format to other figure formats. The svg format is a popular format for vector images. Compared to png, svg can be scaled to any size without losing resolutions. To open a previous flux map, click the button ‘Open a flux map’, and select the desired map.

An example result is shown below.

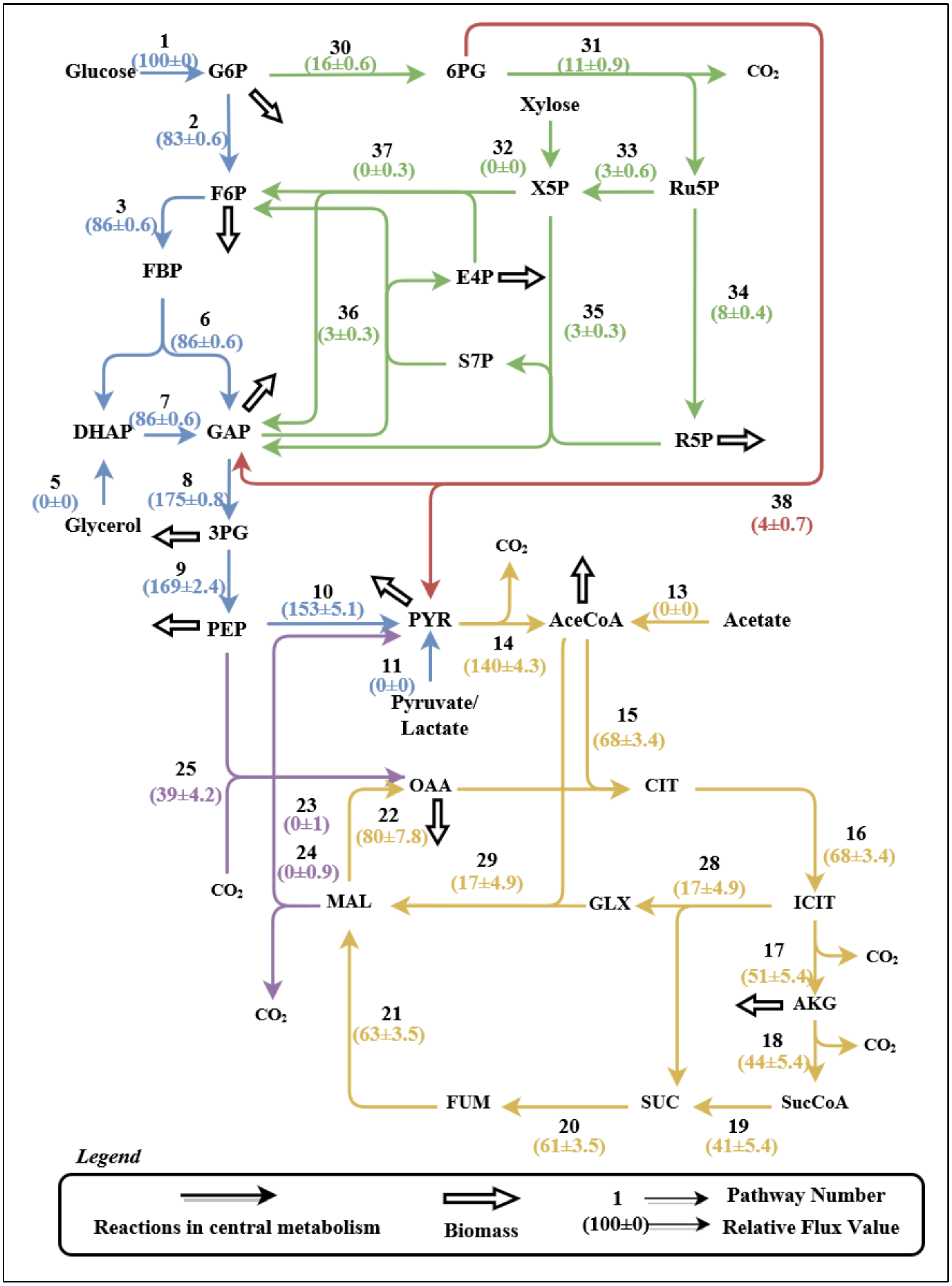
Glucose
G6P
6PG
CO2
Xylose
X5P
Ru5P
F6P
FBP
E4P
S7P
DHAP
GAP
R5P
Glycerol
3PG
PEP
PYR
AceCoA
Acetate
Pyruvate/ Lactate
OAA
CIT
MAL
GLX
ICIT
AKG
SucCoA
SUC
FUM
1 (100±0)
2 (83±0.6)
3 (86±0.6)
5 (0±0)
6 (86±0.6)
7 (86±0.6)
8 (175±0.8)
9 (169±2.4)
10 (153±5.1)
11 (0±0)
13 (0±0)
14 (140±4.3)
15 (68±3.4)
16 (68±3.4)
17 (51±5.4)
18 (44±5.4)
19 (41±5.4)
20 (61±3.5)
21 (63±3.5)
22 (80±7.8)
23 (0±1)
24 (0±0.9)
25 (39±4.2)
28 (17±4.9)
29 (17±4.9)
30 (16±0.6)
31 (11±0.9)
32 (0±0)
33 (3±0.6)
34 (8±0.4)
35 (3±0.3)
36 (3±0.3)
37 (0±0.3)
38 (4±0.7)
Legend
Reactions in central metabolism
Biomass
1 Pathway Number
(100±0) Relative Flux Value

You can generate MID figures comparing the experimental and simulated data by clicking ‘Generate an MID figure’ and then choosing an ion fragment or metabolite. Below is an example result:

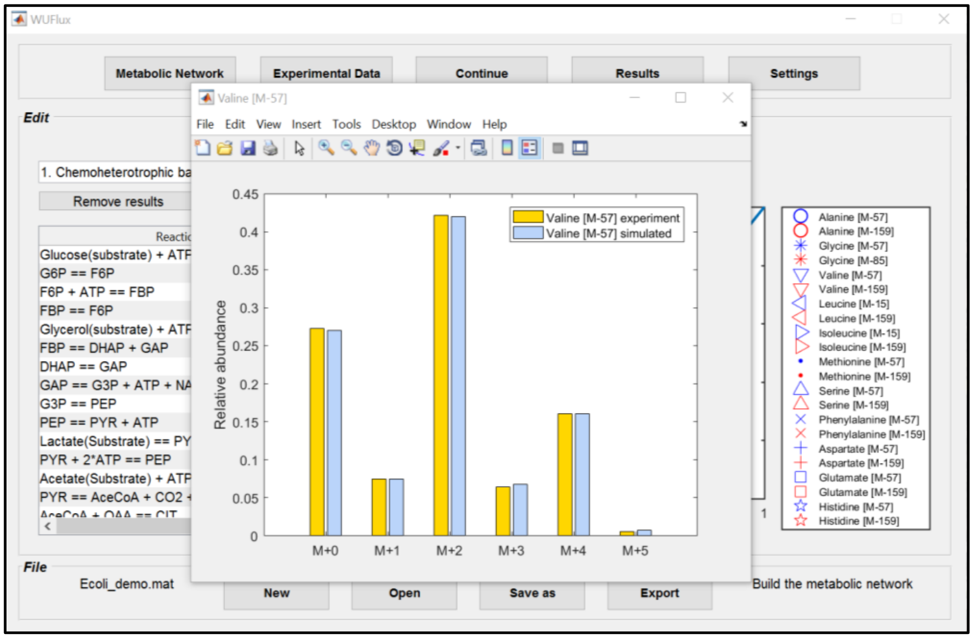

All saved data and results will be stored in the ‘.mat’ format, which can be found in the current folder. They can be loaded into the workspace, and then you can process the data directly in MATLAB. You can export all the results to Excel files by clicking the ‘Export’ button. The file includes information such as the flux distribution, metabolic network information, and a comparison between the experimental MID data and the simulated MID data.

Appendix 1. Description of files in WUFlux

File Name	File Description/ Function
**WUFlux.m**	Main file of WUFlux.
**WUFlux.fig**	Designing the interface of WUFlux.
**EMU_Chemoheterotroph_bacteria**	Calculating the flux distribution of *E. coli* or similar species.
**EMU_Cyanobacteria**	Calculating the flux distribution of *Synechocystis* 6803 or similar species.
**EMU_Vanillin**	Calculating the flux distribution of *Sphingobium* sp. SYK-6 or similar species.
**popcallback.m**	Metabolic reactions for different templates.
**subEMU.m**	Generating EMUs of labeled substrates.
**edit1callback.m**	Editing the non-labeled biomass ratio.
**uitable1callback.m**	Editing metabolic network.
**uitable2callback.m**	Editing linear equality/inequality constraints.
**uitable3callback.m**	Editing MID data.
**uitable4callback.m**	Editing substrate labeling information.
**uitable5callback.m**	Editing optimization settings.
**uitable7callback.m**	Editing measured flux values.
**button1callback.m**	‘Add a reaction’ button.
**button2callback.m**	‘Undo’ button.
**button3callback.m**	‘Load MID data’ button.
**button4callback.m**	‘Convert Raw MS data’ button.
**button5callback.m**	‘Remove Results’ button.
**button6callback.m**	‘Generate a flux map’ button.
**button7callback.m**	‘Generate an MID figure’ button.
**button8callback.m**	‘Open a flux map’ button.
**Demo_E.coli.mat**	Demo for flux analysis of an *E. coli* model.
**Demo_Synechocystis.mat**	Demo for flux analysis of a *Synechocystis* 6803 model.
**Demo_Shewanella.mat**	Demo for flux analysis of a *Shewanella* model.
**Demo_Pseudomonas.mat**	Demo for flux analysis of a *Pseudomonas* model.
**Cyanobacteria_template.svg***	Template for the flux map of cyanobacterial species.
**Ecoli_template.svg***	Template for the flux map of chemoheterotrophic bacteria species.
**Vanillin_template.svg***	Template for the flux map of vanillin-degrading bacteria species.
**MIDtemplate_corrected.xlsx***	Excel template for corrected MID data.
**MIDtemplate_uncorrected.xlsx***	Excel template for uncorrected MID data or raw MS data (for only TBDMS-derivatized proteinogenic amino acids).

*: Do not change the template styles, otherwise WUFlux may not recognize the data properly.

Appendix 2. Abbreviations for metabolites

Abbreviations	Full Name	Abbreviations	Full Name
**G3P***	3-phosphoglycerate	**ICIT**	isocitrate
**PG6***	6-phosphogluconate	**ILE**	isoleucine
**AceCoA**	acetyl-CoA	**LEU**	leucine
**AKG**	α-ketoglutarate	**MAL**	malate
**ALA**	alanine	**OAA**	oxaloacetate
**ARG**	arginine	**PEP**	phosphoenolpyruvate
**ASN**	asparagine	**PHE**	phenylalanine
**CIT**	citrate	**PRO**	proline
**CO_2**	carbon dioxide	**PYR**	pyruvate
**CYS**	cysteine	**R5P**	ribose 5-phosphate
**DHAP**	dihydroxyacetone phosphate	**Ru5P**	ribulose 5-phosphate
**E4P**	erythrose 4-phosphate	**RuBP**	ribulose 1,5-bisphosphate
**F6P**	fructose 6-phosphate	**S7P**	sedoheptulose 7-phosphate
**FBP**	fructose 1,6-bisphosphate	**SBP**	sedoheptulose 1,7-bisphosphate
**FUM**	fumarate	**SER**	serine
**G6P**	glucose 6-phosphate	**SUC**	succinate
**GAP**	glyceraldehyde 3-phosphate	**SucCoA**	succinyl-CoA
**GLC**	glycolate	**THR**	threonine
**GLN**	glutamine	**THF**[#]	tetrahydrofolate
**GLX**	glyoxylate	**TRP**	tryptophan
**GLY**	glycine	**VAL**	valine
**GLU**	glutamate	**X5P**	xylulose 5-phosphate
**HIS**	histidine		

*: The name of symbolic variables in MATLAB cannot start with a number, so we change the regular abbreviations of 3PG and 6PG to G3P and PG6, respectively.

[#]: C1 units include methyl-THF, methylene-THF, and formyl-THF.
