# Supplementary material for: WUFlux: an open-source platform for 13C metabolic flux analysis of bacterial metabolism
Source: BMC Bioinformatics. 2016 Nov 4;17:444. doi: 10.1186/s12859-016-1314-0 (PMC5096001; doi:10.1186/s12859-016-1314-0)
Supplement: Additional file 2: — Comparison of flux estimations from WUFlux, METRAN, and INCA. (DOCX 18 kb) [file 12859_2016_1314_MOESM2_ESM.docx]

| **Pathway** | **E. coli Strains** | **Control Strain** | | | **Engineered Strain** | | |
| --- | --- | --- | --- | --- | --- | --- | --- |
|  | **Software** | **WUFLUX** | **METRAN** | **INCA** | **WUFLUX** | **METRAN** | **INCA** |
| **Glycolysis** | **G6P == F6P** | **69(70)** | **71** | **69** | **55(56)** | **57** | **55** |
|  | **F6P + ATP == FBP** | **80(82)** | **81** | **80** | **77(80)** | **78** | **78** |
|  | **GAP == 3PG + ATP + NADH** | **166(169)** | **167** | **166** | **170(174)** | **171** | **170** |
|  | **3PG== PEP** | **151(155)** | **152** | **151** | **162(168)** | **163** | **162** |
|  | **PEP == PYR + ATP** | **120(120)** | **121** | **119** | **147(153)** | **148** | **148** |
|  | **6PG == PYR + GAP** | **2(1)** | **2** | **1** | **5(3)** | **4** | **5** |
| **TCA cycle** | **PYR == AceCoA + CO_2_ + NADH** | **100(107)** | **101** | **99** | **139(152)** | **141** | **141** |
|  | **AceCoA + OAA == CIT** | **27(37)** | **29** | **28** | **21(33)** | **22** | **21** |
|  | **CIT == ICIT** | **27(37)** | **29** | **28** | **21(33)** | **22** | **21** |
|  | **ICIT == AKG + CO_2_ + NADPH** | **26(35)** | **27** | **27** | **19(29)** | **20** | **18** |
|  | **AKG == SucCoA + CO_2_ + NADH** | **16(26)** | **17** | **18** | **14(26)** | **15** | **13** |
|  | **SucCoA == SUC + ATP** | **12(22)** | **13** | **14** | **12(24)** | **13** | **11** |
|  | **SUC == FUM + FADH2** | **18(28)** | **19** | **19** | **16(29)** | **17** | **16** |
|  | **FUM == MAL** | **21(31)** | **23** | **22** | **18(30)** | **19** | **18** |
|  | **MAL == OAA + NADH** | **23(26)** | **23** | **22** | **20(29)** | **21** | **20** |
| **Pentose phosphate pathway** | **G6P == 6PG + NADPH** | **29(29)** | **28** | **30** | **44(43)** | **42** | **44** |
|  | **6PG == CO_2_ + Ru5P + NADPH** | **27(28)** | **26** | **27** | **38(40)** | **38** | **39** |
|  | **Ru5P == X5P** | **12(13)** | **11** | **12** | **22(24)** | **22** | **22** |
|  | **Ru5P == R5P** | **15(15)** | **15** | **15** | **16(16)** | **16** | **16** |
|  | **X5P + R5P == GAP + S7P** | **7(8)** | **7** | **7** | **12(13)** | **12** | **12** |
|  | **GAP + S7P == E4P + F6P** | **7(8)** | **7** | **7** | **12(13)** | **12** | **12** |
|  | **X5P + E4P == GAP + F6P** | **4(5)** | **4** | **4** | **10(11)** | **10** | **10** |

**Table S1. Comparison of flux estimations derived from WUFlux, METRAN, and INCA.**

Note: The fluxes in parentheses were calculated without consideration of 99% isotopic purity of ^13^C-labeled carbons nor 1.07% ^13^C natural abundance.
